# An Aspartate-Specific Solute-Binding Protein Regulates Protein Kinase G Activity To Control Glutamate Metabolism in Mycobacteria

**DOI:** 10.1128/mBio.00931-18

**Published:** 2018-07-31

**Authors:** Nabanita Bhattacharyya, Irene Nailain Nkumama, Zaccheus Newland-Smith, Li-Ying Lin, Wen Yin, Rebecca E. Cullen, Jack S. Griffiths, Alexander R. Jarvis, Michael J. Price, Pei Ying Chong, Russell Wallis, Helen M. O’Hare

**Affiliations:** a Leicester Tuberculosis Research Group, Department of Infection, Immunity and Inflammation, University of Leicester, Leicester, United Kingdom; b Leicester Drug Discovery and Diagnostics, University of Leicester, Leicester, United Kingdom; c Department of Molecular and Cell Biology, University of Leicester, Leicester, United Kingdom; Institut Pasteur

**Keywords:** *Actinobacteria*, *Corynebacterium*, Mycobacterium tuberculosis, sensory transduction processes, serine/threonine kinases, structural biology

## Abstract

Signaling by serine/threonine phosphorylation controls diverse processes in bacteria, and identification of the stimuli that activate protein kinases is an outstanding question in the field. Recently, we showed that nutrients stimulate phosphorylation of the protein kinase G substrate GarA in Mycobacterium smegmatis and Mycobacterium tuberculosis and that the action of GarA in regulating central metabolism depends upon whether it is phosphorylated. Here we present an investigation into the mechanism by which nutrients activate PknG. Two unknown genes were identified as co-conserved and co-expressed with PknG: their products were a putative lipoprotein, GlnH, and putative transmembrane protein, GlnX. Using a genetic approach, we showed that the membrane protein GlnX is functionally linked to PknG. Furthermore, we determined that the ligand specificity of GlnH matches the amino acids that stimulate GarA phosphorylation. We determined the structure of GlnH in complex with different amino acid ligands (aspartate, glutamate, and asparagine), revealing the structural basis of ligand specificity. We propose that the amino acid concentration in the periplasm is sensed by GlnH and that protein-protein interaction allows transmission of this information across the membrane via GlnX to activate PknG. This sensory system would allow regulation of nutrient utilization in response to changes in nutrient availability. The sensor, signaling, and effector proteins are conserved throughout the *Actinobacteria*, including the important human pathogen Mycobacterium tuberculosis, industrial amino acid producer Corynebacterium glutamicum, and antibiotic-producing *Streptomyces* species.

## IMPORTANCE

Tuberculosis (TB) kills 5,000 people every day, and the prevalence of multidrug-resistant TB is increasing in every country. The processes by which the pathogen Mycobacterium tuberculosis senses and responds to changes in its environment are attractive targets for drug development. Bacterial metabolism differs dramatically between growing and dormant cells, and these changes are known to be important in pathogenesis of TB. Here, we used genetic and biochemical approaches to identify proteins that allow M. tuberculosis to detect amino acids in its surroundings so that it can regulate its metabolism. We have also shown how individual amino acids are recognized. The findings have broader significance for other actinobacterial pathogens, such as nontuberculous mycobacteria, as well as *Actinobacteria* used to produce billions of dollars of amino acids and antibiotics every year.

## INTRODUCTION

Nutrient sensing is central to the ability of bacteria to adapt to changing environmental conditions. Both intracellular and extracellular molecules are sensed in order to regulate fundamental processes such as primary metabolism, motility, and cell division. Multiple mechanisms control gene expression and protein activity in response to these stimuli, and both transcriptional regulation and posttranslational modification are recognized as important (1). Among the many forms of posttranslational modification, protein phosphorylation is ubiquitous (2). Protein kinase G (PknG) stands out among bacterial serine/threonine protein kinases (STPKs) because of its wide conservation in the *Actinobacteria* and the wealth of information on its requirement for virulence of Mycobacterium tuberculosis (3–5) and its role in glutamate overproduction by the industrial organism Corynebacterium glutamicum (6). The downstream effects of PknG activity include well-characterized effects on the activity of enzymes involved in the tricarboxylic acid (TCA) cycle and glutamate metabolism (7, 8).

Identification of stimuli and mechanisms of activation are outstanding questions for bacterial STPKs (9). Based on the striking changes in amino acid metabolism caused by *pknG* disruption in multiple organisms (M. tuberculosis, Mycobacterium smegmatis, and C. glutamicum) (3, 6, 10), we recently tested for nutrient conditions that lead to PknG-dependent phosphorylation of the regulatory protein GarA. GarA was unphosphorylated in M. tuberculosis or M. smegmatis cells that were starved of amino acids, but became phosphorylated when cells were exposed to media containing amino acids (10). Glutamate and aspartate were identified as the stimuli that lead to rapid phosphorylation of GarA (10).

The mechanism of PknG activation by amino acids is unknown. Unlike receptor kinases, such as PknB, where binding of ligands to extracellular domains stimulates intracellular kinase activity, PknG lacks any secretion signal, transmembrane helix, or extracellular domains, so it is unlikely to bind directly to external nutrients. The crystal structure of PknG did not reveal likely amino acid binding sites, implying that activation requires accessory components (11). In addition to its kinase domain, PknG has three further domains: the N-terminal domain is rich in autophosphorylation sites (8), the rubredoxin domain has a metal coordinated by four cysteines (11), and the C-terminal domain is made up of tetratricopeptide repeats (TPRs), a motif that frequently binds peptides or proteins (12). Deletion of these domains had a complex effect on the activity of recombinant PknG, suggesting that they regulate kinase activity by a mix of activatory and repressive functions (13). The activity of recombinant PknG has further been shown to be modulated by the metal-binding site of the rubredoxin domain: activity was enhanced by oxidation (14) and inhibited by nitro-alkylation (15).

In this study, we investigated the mechanism by which amino acids could activate PknG. Our data show that two accessory proteins encoded in the PknG operon are required for PknG activation: GlnX and GlnH. The conserved aspartate-specific solute-binding protein (SBP) GlnH links extracellular amino acid concentration to PknG activity via the periplasmic transmembrane protein GlnX, thereby controlling glutamate metabolism in *Mycobacterium* spp.

## RESULTS

### PknG activation by glutamate is indirect and dependent on its protein interaction domain (TPR domain).

**FIG 1:**
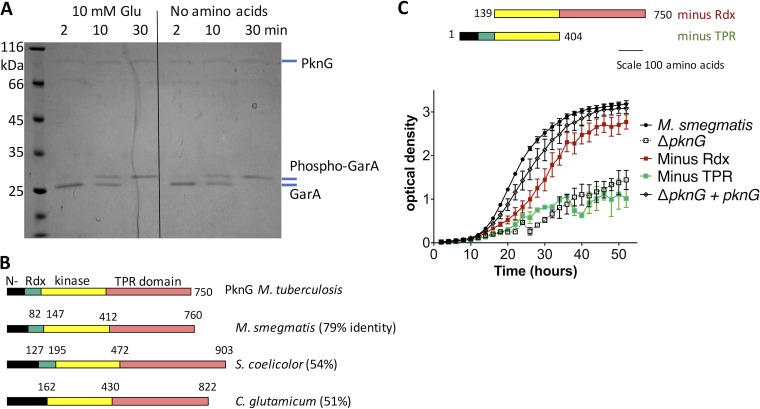
PknG activation by glutamate was indirect, and PknG function required the conserved protein interaction domain (TPR domain). (A) Kinase activity of recombinant PknG toward GarA was independent of glutamate. Kinase reactions in the presence or absence of glutamate were sampled at the indicated times. Phosphorylation of GarA was detected by the known decrease in mobility of phospho-GarA in SDS-PAGE (8, 10). The rate of GarA phosphorylation was similar to that seen in previous studies of PknG and was unchanged by the addition of glutamate. Images are representative of three independent experiments. Similar results were obtained with other putative activators: glutamine, aspartate, and α-ketoglutarate. (B) PknG homologues from *Actinomycetales* have a variable N-terminal domain, sometimes lack the Rdx domain (absent from homologues in the *Corynebacteriaceae*), and always have a tetratricopeptide repeat (TPR) domain. (C) Truncated PknG lacking the Rdx was able to restore the ability of *pknG*-deficient M. smegmatis to grow on minimal medium containing glutamate as the sole nitrogen source, but truncated PknG lacking the TPR domain did not improve growth. Error bars show standard deviations from three wells, and the growth curve is representative of three independent experiments.

Based on the observation that glutamate stimulated PknG to phosphorylate GarA in M. smegmatis cells (10), we tested whether glutamate would increase the rate of phosphorylation of GarA by recombinant PknG (Fig. 1A). The rate of GarA phosphorylation matched previous measurements (8) and was independent of glutamate (Fig. 1A). This correlates with the lack of amino acid binding sites identified by sequence and structural analyses of PknG (11, 13) and led us to hypothesize that glutamate activates PknG via an indirect mechanism, such as interaction with additional proteins. All PknG homologues have a potential protein-protein interaction domain since the conserved C terminus is made up of tetratricopeptide repeats (TPRs) (Fig. 1B), a motif associated with protein-protein interactions (12).

To investigate the domains required for the function of PknG, we used a previously characterized *pknG*-disrupted mutant strain of M. smegmatis (16). This strain grows normally in standard mixed medium, but grows slowly with clumps when glutamate is the sole nitrogen source (10).

Protein domain boundaries were identified from previous structural and biochemical characterization of M. tuberculosis (13). PknG and plasmids were constructed for expression of truncated versions of PknG (Fig. 1C). Expression of all versions of PknG was detected by Western blotting (see Fig. S1A in the supplemental material), and all strains grew normally on mixed medium (Fig. S1B). Full-length PknG and truncation mutants that included the TPR domain improved the growth on glutamate, but truncation mutants that lacked the TPR domain did not improve growth (Fig. 1C). These data suggest that the TPR domain is required for the role of PknG in regulating glutamate metabolism—probably by mediating interactions with other protein(s). Notably the TPR domain is conserved in all PknG homologues, but the N-terminal domain is weakly conserved and the rubredoxin domain is absent from the *Corynebacteriacaea* (Fig. 1B).

10.1128/mBio.00931-18.1 FIG S1  (A) Expression of PknG and truncated PknG in *pknG*-disrupted M. smegmatis was confirmed by Western blotting and probed with anti-PknG antibody. (B) Expression of truncated PknG did not hinder growth of *pknG*-deficient M. smegmatis (growth curves on standard Sauton’s medium). Download FIG S1, JPG file, 0.2 MB. Copyright © 2018 Bhattacharyya et al. 2018 Bhattacharyya et al. This content is distributed under the terms of the Creative Commons Attribution 4.0 International license.

### PknG is encoded in a conserved operon and functionally linked to transmembrane sensor protein GlnX.

**FIG 2:**
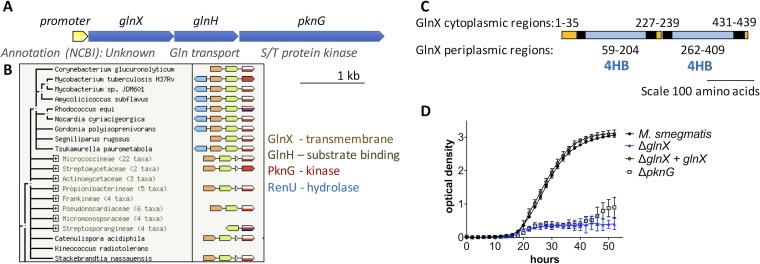
PknG is co-expressed with and functionally linked to putative sensor proteins GlnH and GlnX. (A) The PknG operon of M. tuberculosis has a single promoter, and the three genes are co-expressed (49). (B) The structure of the PknG operon is conserved widely in the *Actinomycetales* (STRING database [42]). (C) The transmembrane segments and membrane topology of GlnX were predicted using TMHMM (38), and putative four-helix bundle sensory domains (4HB) were detected using pfam (39). Putative transmembrane helices are shown in black, cytoplasmic regions in orange, and periplasmic regions in blue. (D) Deletion of *glnX* from M. smegmatis mirrored the phenotype of *pknG*-deficient M. smegmatis: a defect in utilization of glutamate as the sole nitrogen source. Growth of *glnX*-deficient M. smegmatis was restored by reintroduction of *glnX*. Error bars represent standard deviations from three wells, and the growth curve is representative of three independent experiments.

To identify putative interaction partners that could activate PknG, we analyzed the genomic region of *pknG*. The operon structure in M. tuberculosis, M. smegmatis, and C. glutamicum has been described previously: PknG is encoded with a predicted lipoprotein and a predicted transmembrane protein (Fig. 2A) (3, 6). This operon arrangement is conserved in the *Actinomycetales* and other *Actinobacteria* that have PknG homologues (Fig. 2B).

The lipoprotein was named GlnH due to homology with a solute-binding protein involved in glutamine transport by Escherichia coli (26% amino acid identity [17]). However, *glnH* disruption did not affect glutamine uptake by C. glutamicum, suggesting that it has been misclassified (6). Both GlnH and the unknown transmembrane protein GlnX were annotated as putative sensory proteins in the microbial signal transduction database MiST (18). GlnX is predicted to have two sensor-like four-helix bundles in the periplasm (pfam12729 accession no. 4HB_MCP_1), similar to the four-helix bundles of nutrient sensors involved in chemotaxis like the Tar protein of E. coli (Fig. 2C). We speculated that GlnX and GlnH could bind each other, similar to Tar receptor interaction with the solute-binding protein maltose-binding protein (MBP) (19, 20) and trimethylamine *N*-oxide (TMAO)-binding protein with sensor histidine kinase TorS (21). In this arrangement, transmembrane GlnX would link periplasmic GlnH to cytoplasmic PknG, allowing PknG to phosphorylate cytoplasmic GarA. The putative interaction between GlnX and PknG would provide an explanation for the cell envelope localization of PknG (22), despite PknG lacking secretion signals or transmembrane domains (SignalP v4.0 [23]). The cytoplasmic termini of GlnX (35 and 6 amino acids at the N terminus and C terminus, respectively) would be typical of the extended peptide partners often recognized by TPR domains (24, 25).

To test whether GlnX is functionally linked to PknG and a candidate for activating PknG, we constructed an unmarked *glnX* deletion mutant strain of M. smegmatis. The strain copied the phenotype of *pknG*-disrupted M. smegmatis: normal growth on mixed medium, with a specific defect in glutamate utilization that could be restored by reintroduction of plasmid-borne *glnX* (Fig. 2D; see Fig. S2 in the supplemental material). This finding correlates with earlier evidence from C. glutamicum, where *glnX* and *glnH* mutations led to a similar amino acid utilization defect as a *pknG* mutant, although complementation data are not available for the earlier study (6).

10.1128/mBio.00931-18.2 FIG S2  *glnX*-disrupted M. smegmatis grew like parental M. smegmatis on a variety of media, but showed a growth defect with clumping when glutamate was the sole source of nitrogen. Download FIG S2, JPG file, 0.3 MB. Copyright © 2018 Bhattacharyya et al. 2018 Bhattacharyya et al. This content is distributed under the terms of the Creative Commons Attribution 4.0 International license.

### GlnH bound Asp, Glu, and Asn, the same amino acids that stimulate GarA phosphorylation in mycobacteria.

**FIG 3:**
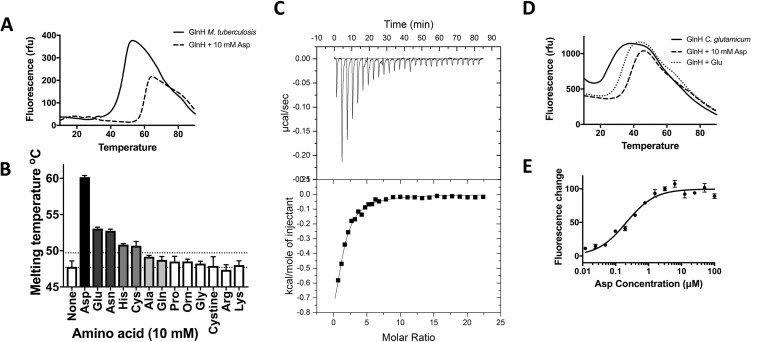
GlnH bound diverse amino acids, with highest affinity for Asp. (A) The melting temperatures of GlnH were 47°C in the absence of amino acids (solid line) and 59°C in the presence of 10 mM aspartate (dashed line). (B) Asp, Glu, Asn, His, and Cys significantly stabilized GlnH (increase in melting temperature of >2°C; *P* < 0.05), whereas other amino acids did not. (C) Binding of Asp to GlnH was measured by isothermal calorimetry (ITC). (D) The melting temperatures of C. glutamicum GlnH were 24°C in the absence of amino acids and 39 or 34°C in the presence of 10 mM Asp or Glu, respectively. (E) Binding of Asp to C. glutamicum GlnH was measured by changes in the intrinsic tryptophan fluorescence. Error bars represent standard deviations from at least three measurements. Graphs are representative of three independent experiments.

GlnH belongs to the solute-binding protein family, specifically the polar amino acid binding family, which bind to arginine, cysteine, histidine, glutamine, glutamate, and aspartate (26). In general, this family is too diverse to predict the ligand specificity of GlnH, so specificity was determined using recombinant protein. Binding of putative ligands to soluble M. tuberculosis GlnH (lacking the secretion and lipoylation sequence in amino acids 1 to 20) was measured by a thermal melt-shift assay, in which protein denaturation is measured in the presence and absence of ligand. Addition of aspartate, glutamate, or asparagine increased the thermal stability of GlnH, suggesting that these are ligands for GlnH (Fig. 3A and B). Addition of cysteine or histidine also led to a modest but statistically significant effect, while other amino acids had no significant effect on the melting temperature. Aspartate led to the greatest stabilization by >10°C. Binding of GlnH and Asp was confirmed by isothermal titration calorimetry (ITC) (Fig. 3C), allowing measurement of the dissociation constant (*K*_*d*_) as 4.8 µM (Table 1). This implies that GlnH might be fully occupied by Asp when M. tuberculosis escapes to the cytoplasm of infected macrophages but is potentially unoccupied in the phagosome. The affinity of GlnH for Asp is relatively weak compared to typical bacterial amino acid transport proteins (*K*_*d*_ = 1 nM to 2 µM [27, 28]), while mammalian *N*-methyl-d-aspartate (NMDA) and metabotropic Glu receptors show a range of affinities for glutamate (50% effective concentration [EC_50_] of 20 nM to 1 mM, although many have EC_50_s of 1 to 10 µM [29, 30]).

**TABLE 1:** Dissociation constants for the proteins examined in this study

Protein	Ligand	*K*_*d*_ (µM)^a^
M. tuberculosis GlnH	Asp	4.8 ± 0.6
	Glu	15.2 ± 5.7
C. glutamicum GlnH	Asp	550 ± 90
	Glu	2,060 ± 390

a Dissociation constants for M. tuberculosis GlnH and Asp or Glu were determined using ITC. Dissociation constants for C. glutamicum GlnH and Asp or Glu were determined using measurements of intrinsic tryptophan fluorescence.

To determine whether the amino acid specificity of the GlnH sensor is conserved in other *Actinobacteria*, we prepared recombinant GlnH from C. glutamicum (43% amino acid identity). The thermal melt-shift assays (Fig. 3D) confirmed that both aspartate and glutamate stabilized GlnH. Furthermore, addition of Asp or Glu to GlnH led to an increase in intrinsic tryptophan fluorescence (Fig. 3E; see Fig. S3 in the supplemental material), revealing that C. glutamicum GlnH binds Asp and Glu with lower affinity than M. tuberculosis GlnH (Table 1). The weaker amino acid binding by C. glutamicum than M. tuberculosis GlnH could reflect differences in the niche and metabolism of these organisms, thus allowing C. glutamicum to respond to changing concentrations of aspartate and glutamate in amino acid-rich environments. C. glutamicum 13032 was originally isolated from soil contaminated with avian feces and was found to secrete up to 75 mM glutamate (now >500 mM in industrial fermentations). Strains of C. glutamicum have also been isolated from diverse environments, including soil, feces, dairy products, plant tissue, and animal skin, some of which are poor and others rich in free amino acids (<5 µM to >100 µM) (31).

10.1128/mBio.00931-18.3 FIG S3  Binding between GlnH and Glu was lower affinity than binding between GlnH and Asp. (A) M. tuberculosis GlnH binding with Glu was measured by ITC. (B) C. glutamicum GlnH binding with Glu was measured by changes in intrinsic fluorescence. Download FIG S3, JPG file, 0.2 MB. Copyright © 2018 Bhattacharyya et al. 2018 Bhattacharyya et al. This content is distributed under the terms of the Creative Commons Attribution 4.0 International license.

### Structure of GlnH.

**FIG 4:**
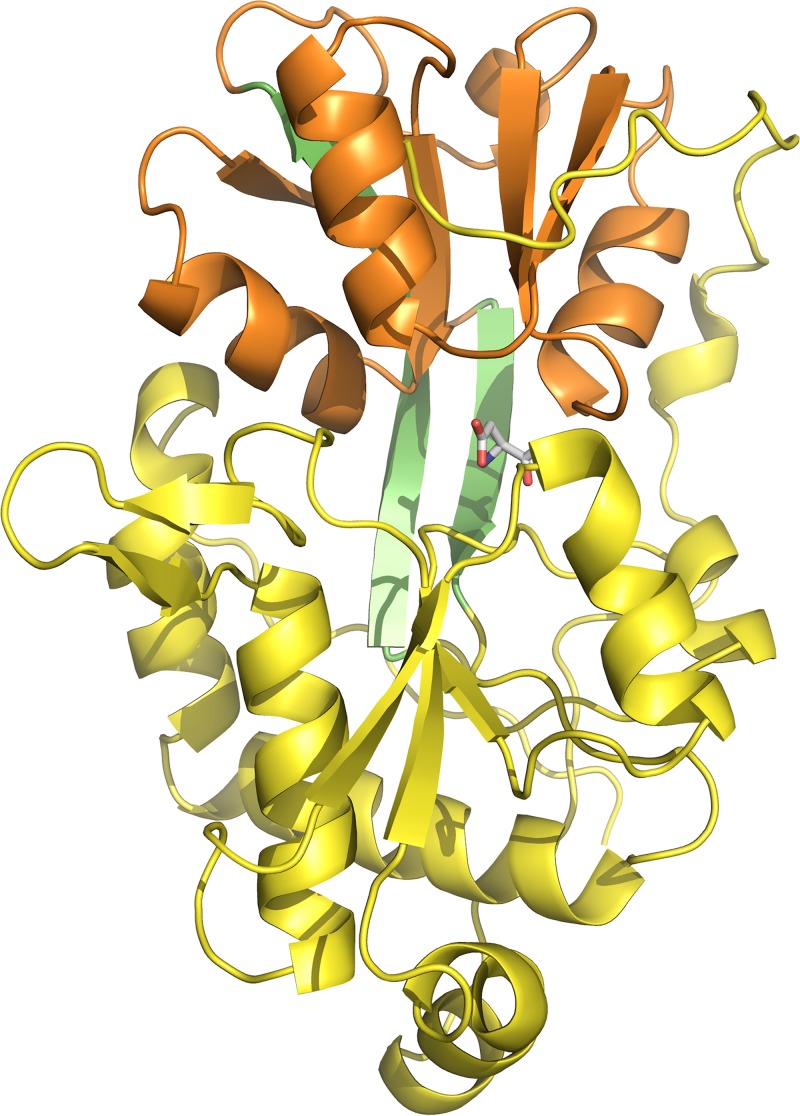
Overall structural presentation of GlnH. GlnH has a Venus flytrap-like structure with two lobes (yellow and orange) joined by a hinge (green). The ligand (stick representation) lies between the lobes and is buried.

To determine how GlnH recognizes its ligands, the structure of GlnH in complex with aspartate was determined by X-ray crystallography. The fold of GlnH is related to bacterial solute-binding proteins from ATP transporters and to the sensor domains of glutamate receptors: two lobes close around the Asp ligand in a Venus flytrap-like arrangement (Fig. 4). Each lobe has an α/β structure with a central parallel β sheet sandwiched by α helices. The Asp ligand is buried at the interface between the two lobes and makes contacts with both lobes.

**FIG 5:**
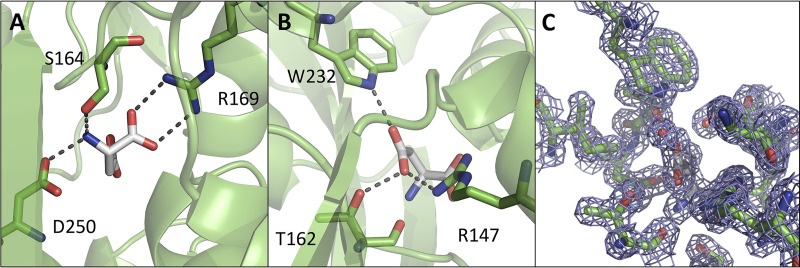
The aspartate binding pocket of GlnH. Two views are presented of the ligand-binding pocket, focused on polar contacts between GlnH and the amino and carboxyl groups of the Asp ligand (A) and the R group of the Asp ligand (B). Side chains that make polar contacts with the Asp ligand are shown in stick mode, with hydrogen bonds and salt bridges shown as broken lines. Asp ligand is shown in white, and GlnH is in green. (C) An equivalent view of the aspartate binding pocket with the electron density map displayed on the Asp ligand, water, and selected protein residues (2*F*_o_ − *F*_c_ map contoured at 1.6 σ).

**FIG 6:**
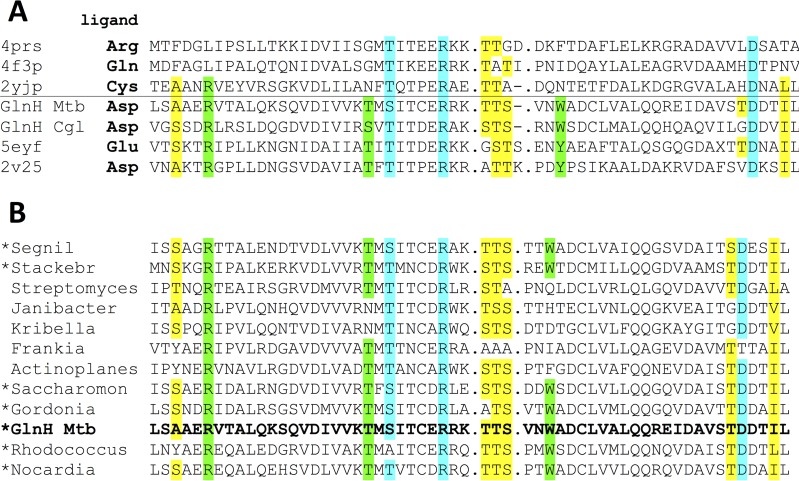
Comparison of the GlnH ligand binding pocket with other amino acid binding proteins. (A) Alignment of GlnH with solute-binding proteins in the PDB showed that the residues whose side chains make polar contact with the R group of the Asp ligand are unique to Asp/Gly-specific solute-binding proteins (highlighted in green), while the residues that contact other parts of the ligand are conserved in solute-binding proteins specific for diverse amino acids. (Side-chain-mediated polar contacts are highlighted in cyan, and residues making hydrophobic or main chain contacts are highlighted in yellow.) Mtb, M. tuberculosis; Cgl, C. glutamicum. (B) Alignment of SBPs encoded with homologues of PknG allowed a prediction of those that are likely to be specific for Asp/Glu (starred).

The amine and carboxyl groups of the Asp ligand are charge neutralized and stabilized by the side chains of Ser 164, Arg 169, and Asp 250 (Fig. 5A), which are conserved in structurally related amino acid binding proteins of diverse specificities (Fig. 6A) (26, 32). The side-chain carboxyl group of the Asp ligand makes hydrogen bonds with the side chains of Arg 147, Trp 232, and Thr 162 (Fig. 5B and C), and these are conserved in solute-binding proteins that have the same ligand specificity as GlnH (Fig. 6B; see Fig. S4 in the supplemental material). The side chain of the Asp ligand also contacts Lys 161 via water.

10.1128/mBio.00931-18.4 FIG S4  Comparison of GlnH with bacterial Asp- and Glu-specific solute-binding proteins. (A) The complex of GlnH with aspartate was aligned with an aspartate binding protein, Peb1a. (Peb1a is wheat colored [PDB accession no. 2V25; RMSD = 2.173 A over 1,201 atoms].) (B) The orientation of the Asp ligand and some of the side-chain-mediated contacts with the ligand are conserved between GlnH and Peb1a. Resides that contact the ligand were identified using PISA and rendered in stick mode. Peb1a is yellow (orange ligand), and GlnH is cyan (blue ligand). (C to E) The complex of GlnH with glutamate was aligned with a glutamate binding protein from Enterococcus faecium (5eyF [RMSD = 1.305 A over 1,061 atoms]). A view of the ligand binding pocket is presented for 3eyF (C), GlnH (D), and the superposition (E). Download FIG S4, JPG file, 0.4 MB. Copyright © 2018 Bhattacharyya et al. 2018 Bhattacharyya et al. This content is distributed under the terms of the Creative Commons Attribution 4.0 International license.

**FIG 7:**
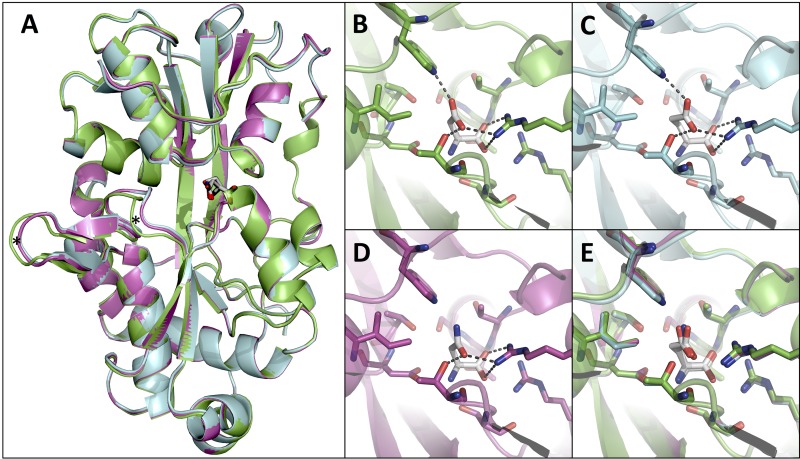
Structural basis of GlnH specificity for Asp. (A) Alignment of GlnH complexes with Asp, Glu, and Asn revealed similar overall structures. (Protein structures were colored according to the ligand: green, cyan, and magenta for the Asp-, Glu-, and Asn-bound proteins, respectively. Ligands were colored by atom type: carbon, white; oxygen, red; nitrogen, blue.) Asterisks highlight two loops that are displaced by >2 A in GlnH-Asp compared to GlnH-Glu or GlnH-Asn. (B to E) Comparison of the ligand binding sites of the aligned structures, with dashed lines indicating the hydrogen bonds and salt bridges between GlnH and Asp (B), Glu (C), and Asn (D).

To determine the structural basis of discrimination between higher- and lower-affinity ligands, GlnH was crystallized in the presence of Glu and Asn. Overall, the structures with different ligands bound were similar (Fig. 7A; root mean square deviation [RMSD] = 0.199 Å over 1,727 main-chain atoms). The greatest difference is in loops 99 to 103 and 109 to 113 (DIGSN and DPITG, respectively), which are displaced 2 Å in the Glu- or Asn-bound structures compared to the Asp-bound structure (Fig. 7A). The conformations of the ligand binding site and positioning of the ligands are similar (Fig. 7B to E). Asn is unable to form a hydrogen bond with Trp 232, providing an explanation for the difference in affinity of binding (Fig. 3B and 7D). Contacts between GlnH and Glu are the same as those between GlnH and Asp, with the liganded Glu adopting a bent conformation such that the side-chain carboxyl group occupies the equivalent position to the side chain of ligand Asp in the GlnH-Asp complex (Fig. 7D). The bent conformation of the Glu ligand is similar to that observed in another bacterial solute-binding protein from Enterococcus faecium (5eyf [Fig. S4]), but different from the extended conformation of Glu seen in mammalian glutamate receptors (see Fig. S5 in the supplemental material). Overall, there is greater conservation of ligand contacts between GlnH and bacterial ABC transporter proteins (Peb1a with Asp and 2eyf with Glu) than with eukaryotic glutamate receptor proteins with glutamate (Fig. S4 and S5).

10.1128/mBio.00931-18.5 FIG S5  Comparison of GlnH with the sensor domains of mammalian glutamate receptors. (A) The structure of GlnH bound to glutamate was aligned with the Glu-binding domain of GluR3 AMPA receptor (PDB accession no. 3DLN [RMSD = 1.725 A over 692 atoms]). Panels B to D present a comparison of the ligand binding site, highlighting the extended conformation of Glu ligand in the mammalian receptor (B), compared to the bent conformation in GlnH (C and overlay in panel D). (E) The structure of GlnH bound to glutamate was aligned with the GluR6 ligand binding core in complex with glutamate (PDB accession no. 1S7Y [RMSD = 1.902 A over 810 atoms]). GlnH and its ligand are shown in cyan, while mammalian glutamate receptors are shown in blue. Download FIG S5, JPG file, 0.4 MB. Copyright © 2018 Bhattacharyya et al. 2018 Bhattacharyya et al. This content is distributed under the terms of the Creative Commons Attribution 4.0 International license.

Sequence alignment of GlnH homologues that are also genetically linked to PknG (Fig. 6B) shows that within the order *Actinomycetales* (which includes important human and animal pathogens like Mycobacterium leprae and Rhodococcus equi), conservation of the residues that contact the R group of the Asp/Glu ligand predicts that these proteins are also likely to be specific for Asp and Glu.

Conformational change of SBP proteins is integral to their function as sensors, with unliganded proteins having a flexible conformation and ligands typically stabilizing a closed conformation, called a “Venus flytrap mechanism” (26). Our structures of liganded GlnH represent “closed” conformations. We used limited trypsin proteolysis to investigate whether unliganded GlnH is more flexible (and therefore susceptible to proteolysis) than GlnH with Asp. The results suggest that binding of Asp provokes conformational change in GlnH (see Fig. S6 in the supplemental material).

10.1128/mBio.00931-18.6 FIG S6  Binding of Asp to GlnH induced conformational change that protected GlnH from trypsin digestion. Eight micrograms of GlnH was incubated with the indicated amount of trypsin in PBS at 20°C for 16 h, in the presence or absence of 1 mM Asp. Reactions were analyzed by SDS-PAGE followed by staining with Coomassie blue. Download FIG S6, JPG file, 0.2 MB. Copyright © 2018 Bhattacharyya et al. 2018 Bhattacharyya et al. This content is distributed under the terms of the Creative Commons Attribution 4.0 International license.

## DISCUSSION

**FIG 8:**
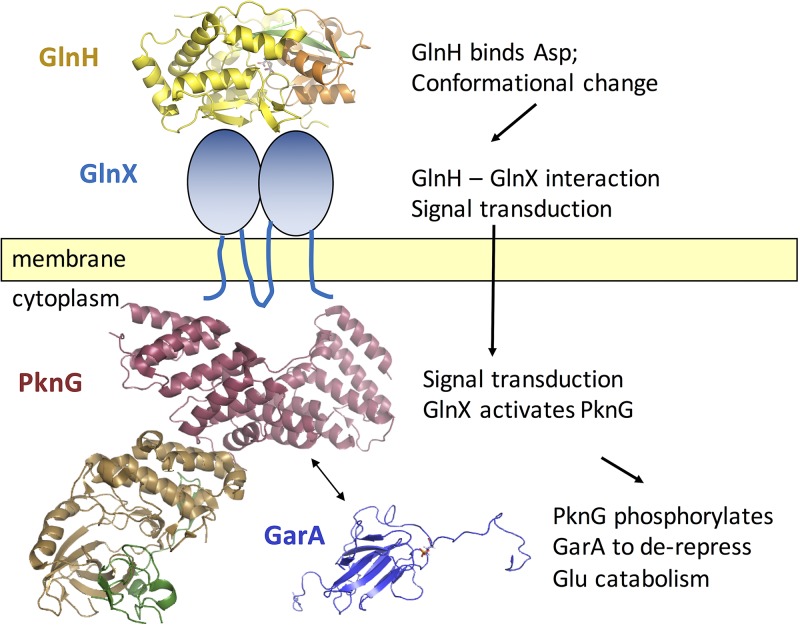
Model of sensing of periplasmic aspartate by GlnH: Asp leads to activation of PknG and derepression of the TCA cycle. In the absence of extracellular amino acids, GarA inhibits catabolism of glutamate through the TCA cycle. In contrast, the presence of aspartate causes conformational change of the sensor GlnH, leading to conformational change of GlnX that activates PknG. PknG becomes autophosphorylated and phosphorylates GarA, thus relieving inhibition of the TCA cycle.

We have identified a genetic and functional linkage between PknG, GlnX, and GlnH and demonstrated a correlation between the amino acids that bind GlnH and those that stimulate PknG phosphorylation of GarA. This suggests a model of sensing and signal transduction by which GlnH and GlnX could activate PknG in response to aspartate or glutamate (Fig. 8). Thus, the presence of amino acids would switch enzyme regulation to allow the organism to degrade amino acids through the TCA cycle.

The mechanism of PknG activation is thought to involve two processes: autophosphorylation of the N-terminal domain, which provides binding sites to recruit the substrate GarA, and displacement of the N-terminal and rubredoxin domains, which restrict substrate access to the catalytic site (13). This is compatible with our model, in which GlnX interacts with the TPR domain to induce activation of conformational changes in PknG. Although the TPR domain is located 20 Å from the catalytic site (11), there are direct interactions between the N- and C-terminal domains of PknG (11, 13) (see Fig. S7 in the supplemental material) that may stabilize the inhibitory position of the rubredoxin domain. GlnX could thus trigger activation by altering the repressive positioning of the rubredoxin domain to allow substrate access and autophosphorylation.

10.1128/mBio.00931-18.7 FIG S7  Structure of PknG (PDB accession no. 2PZI) showing the TPR domain (gray surface) connected to the catalytic domain (gray cartoon) by a blue linker. Interdomain interactions between the linker and truncated N terminus (red) mean that ligand binding to the TPR domain could affect the positioning of the repressive N-terminal and rubredoxin domains and thus regulate access of substrates to the active site. The rubredoxin domain (cyan) of PknG restricts access to the active site. (Inhibitor bound at the active site is shown in yellow.) The active site is 20 Å (long arrow) from the TPR repeats of PknG, but there are direct interactions (short arrow) between the N-terminal domain (red) and the linker. Residues that are conserved in the TPR domains of PknG homologues are colored magenta and are mainly clustered in a groove in the concave face of the TPR domain and at the C-terminal tip. Download FIG S7, JPG file, 0.2 MB. Copyright © 2018 Bhattacharyya et al. 2018 Bhattacharyya et al. This content is distributed under the terms of the Creative Commons Attribution 4.0 International license.

The TPR domain of PknG homologues has a variable number of TPRs with weak sequence conservation. Only 36 of the 345 residues are invariant, and 20 of these line a groove in the concave face (Fig. S7), with another cluster at the C-terminal tip of the TPR domain. These surfaces represent candidate binding sites for GlnX: indeed, the concave face is the binding site for peptide ligands of TPR domains of other proteins. In the PknG inhibitor structure (11), the groove is occupied by a linker that connects the catalytic and TPR domains. GlnX might displace this linker, which would disrupt the interactions that link the N and C termini of PknG and hence displace the rubredoxin domain. However, further structures of full-length PknG and complexes of PknG with GlnX will be required to determine the structural changes associated with kinase activation.

Our model would represent a new role for the SBP family as sensors for serine/threonine protein kinases, in addition to the known roles in solute transport and as sensors for histidine kinases, G protein-coupled receptors, and ion channels (33). The model applies to many medically and industrially important bacteria, since the genetic linkage of GlnH with PknG is widely conserved in the *Actinobacteria*. The GlnH structures and sequence alignments allow us to predict that all PknG-linked GlnH homologues bind amino acids, and those within the order *Actinomycetales* are likely to be specific for Asp/Glu.

There is conflicting evidence about the subcellular localization of PknG: it has been detected in cytoplasm and cell envelope of M. tuberculosis (34), cell envelope of Mycobacterium marinum (22), and the cytosol of macrophages infected with Mycobacterium bovis BCG (4). Association of PknG with the membrane of mycobacteria might reflect the interaction with GlnX. The possibility that PknG shuttles between the periplasmic space and the cytoplasm to sense local amino acid concentrations directly can be excluded because PknG alone is insensitive to amino acids (Fig. 1); rather it requires GlnH and GlnX to transmit information across the inner membrane of the cell to the cytoplasmic face, where PknG phosphorylates GarA (located in the cytoplasm).

Other stimuli of PknG activity apart from amino acids have been reported: oxidative stress sensed by the rubredoxin domain (14). Stimuli that change the structure of the rubredoxin domain could remove the restriction on substrate access to the active site (13). In our experiments using M. smegmatis in minimal medium, we found that amino acids were the strongest stimulus for phosphorylation (10), but additional factors also influenced phosphorylation to a lesser extent, suggesting that PknG may respond to multiple stimuli. The multidomain structure of PknG makes it a plausible candidate to integrate multiple signals. GlnX is another candidate sensor for additional sensory inputs as it is homologous to the E. coli Tar receptor, which binds both protein and small molecule activators (19, 20). Fitting with its activation by multiple stimuli, PknG also has multiple substrates and may regulate other processes in addition to regulation of the TCA cycle via GarA (5, 35).

TPR domains have been identified in kinases from multiple bacterial phyla (>1,000 listed in Interpro [36]), suggesting that TPR-mediated protein interaction could be a widespread regulatory mechanism of bacterial serine/threonine protein kinases. Activation of serine/threonine protein kinases by solute-binding proteins may also be widespread, as there are further examples beyond PknG of serine/threonine protein kinases genetically linked to solute-binding proteins (for example, Streptomyces griseus SGR_4432 and Streptomyces coelicolor SCO1468), and also genes that encode both kinase and SBP domains in a single polypeptide. (MSMEG_1200 is an example that is conserved in multiple *Mycobacterium* and *Streptomyces* species; Interpro lists a further 260 SBP kinases.) PknG stands out from the examples mentioned above by its wide conservation in diverse bacteria.

## MATERIALS AND METHODS

### PknG activity assay.

GarA phosphorylation was detected by the change in mobility of phosphorylated GarA in SDS-PAGE, as previously (8, 10). Recombinant hexahistidine-tagged PknG and GarA of M. tuberculosis were purified as described previously (8). Reaction mixtures contained 175 nM PknG, 7.7 mM GarA, 25 mM Tris-HCl (pH 7.4), 5 mM MgCl_2_, 2 mM MnCl_2_ and 0 or 10 mM glutamate (sodium salt). Reactions were started by the addition of 0.1 mM ATP, and mixtures were incubated at 37°C. Aliquots were taken after 2, 10, and 30 min, stopped by addition of 1% SDS, and analyzed by SDS-PAGE with Coomassie staining. Images are representative of three independent experiments.

### Growth of M. smegmatis.

M. smegmatis wild-type strain mc^2^155 and mutant strains were cultured in Middlebrook 7H9 broth supplemented with 0.05% Tween 80 and 10% ADN (0.5% bovine serum albumin, 0.2% dextrose, 0.085% NaCl) and Middlebrook 7H10 agar with 10% ADN. Middlebrook provides a mixture of carbon and nitrogen sources (glycerol, glucose, NH_4_Cl, glutamate, and Tween). When required, antibiotics were added: 100 µg/ml hygromycin and 30 µg/ml kanamycin.

### Growth assay for the ability of truncated PknG to complement *pknG*-deficient M. smegmatis.

The *pknG*-disrupted (Δ*pknG*) M. smegmatis mutant, containing the hygromycin resistance cassette (16), was previously shown to be deficient in glutamate utilization (16). Plasmid-based expression of either M. smegmatis or M. tuberculosis PknG restored the defect (16). Truncated PknG was cloned in the EcoRI/HindIII sites of pMV261 and verified by sequencing. (pMV261 is an episomal shuttle vector with a kanamycin resistance cassette and an *hsp60* promoter for constitutive expression of the introduced genes [37].) Plasmids were introduced to the M. smegmatis Δ*pknG* mutant by electroporation. Expression was verified by Western blotting using an antibody raised against recombinant M. tuberculosis PknG (Cambridge BioScience) and anti-rabbit horseradish peroxidase (HRP) (Sigma). These strains of M. smegmatis were tested for their growth on standard mixed medium (7H9) or minimal medium containing glutamate (10 mM) as the sole nitrogen source as described previously (10).

### Protein sequence analysis.

Analysis of the amino acid sequence of PknG, GlnX, GlnH, and homologues used TMHMM (38), pfam (39), Clustal Omega (40, 41), and STRING (42).

### Construction of the unmarked M. smegmatis *glnX* mutant.

An in-frame, unmarked *glnX* deletion mutant was constructed by homologous recombination, using a published method (43). Counterselection (2% sucrose and 80 µg ml^−1^ X-Gal [5-bromo-4-chloro-3-indolyl-β-d-galactopyranoside]) was used to identify putative double-crossover unmarked deletion mutants, which were verified by PCR. A complementation vector was constructed by cloning M. smegmatis *glnX* with its cognate promoter into pMV306. pMV306 is an integrative plasmid in which the expression cassette of pMV361 has been replaced by a multiple cloning site (37).

### Expression and purification of GlnH.

The signal sequence of GlnH, as identified using SignalP (10), is followed by a lipobox motif (residues 23 to 26 [LASC]). The region encoding the predicted soluble domain of GlnH (codon 27 onwards) was amplified from genomic DNA of M. tuberculosis H37Rv (forward primer TACTTCCAATCCATGGGCCACTCGGAAACGCTG and reverse primer TATCCACCTTTACTGTCAGTCCACATACCTCGGCGT) and cloned inserted into an expression vector derived from pET-43.1a(+) (Novagen) fused with an N-terminal His_6_ tag (BD In-Fusion PCR cloning kit; Clontech). Sequence-verified plasmid was transformed into Escherichia coli Shuffle cells (New England Biolabs) and grown on Luria agar (LA) with 100 µg/ml ampicillin at 30°C. Transformed cells were grown in Luria broth (LB) with 100 µg/ml ampicillin at 30°C with shaking. Expression was induced using 0.2 mM IPTG (isopropyl-β-d-thiogalactopyranoside) at 20°C for 18 h. GlnH was purified using Ni-nitrilotriacetic acid (NTA) resin in 50 mM Tris-HCl (pH 7.5)–150 mM NaCl and then by size exclusion chromatography in 20 mM Tris-acetate (pH 7.5)–20 mM NaCl. GlnH eluted from Superdex 75 (16/60) in the fractions corresponding to the predicted size of monomeric protein (35,455 Da, eluting in fractions at 62 to 64 ml). Protein was concentrated to 8 mg/ml and stored at −80°C.

### Expression and purification of GlnHcg.

The gene encoding the soluble domain of the GlnH homologue from C. glutamicum (GlnHcg) was amplified from genomic DNA of C. glutamicum ATCC 13032 using primers GlnHcg1 and GlnHcg2 (AACATATGACTCCAACACCTGTGGAACC and TTCTCGAGTTATCCTTCATCGTTTTCTGTC, respectively) and cloned into the NdeI/XhoI sites of pET15b (Novagen), giving an N-terminal His_6_ tag. The protocol for protein expression and purification was identical to that of GlnH, with the exception that phosphate-buffered saline (PBS) was used for metal affinity purification.

### Measurement of the effects of amino acids on GlnH thermal stability.

Amino acids were chosen for testing as ligand candidates by examining the ligands of the most closely related structures in the PDB (solute-binding protein cluster F4 [26]): Asp, Glu, Asn, Gln, Cys, His, Arg, and Lys, as well as the controls Ala and Gly.

GlnH or GlnHcg (0.15 mg/ml) was mixed on ice with SyPro orange dye (Sigma-Aldrich) diluted 1:10,000 in 100 mM Tris-acetate (pH 7.5), and putative ligands were added to 10 mM. Using a quantitative PCR (qPCR) machine, fluorescence was recorded as the temperature was increased from 4 to 90°C (excitation, 470 nm; emission, 570 nm). The temperature at the midpoint of thermal denaturation was determined by taking the first differential of fluorescence versus temperature in the transition (greatest rate of change of fluorescence).

### Measurement of amino acid binding to GlnH by isothermal titration calorimetry.

Binding of Asp and Glu to GlnH was measured by microcalorimetry using a VP-ITC microcalorimeter (MicroCal) with GlnH at 20 mM in 100 mM HEPES (pH 7)–50 mM NaCl and injections of 2 mM Asp or Glu into the same buffer.

### Measurement of the effects of amino acids on intrinsic tryptophan fluorescence of GlnH.

Intrinsic fluorescence (excitation, 292 nm; emission, at 340 nm) of GlnHcg (0.15 mg/ml) was measured in phosphate-buffered saline at a range of concentrations of amino acid ligand. The dissociation constant of GlnH for amino acids was calculated by fitting fluorescence data to a one-site binding equation.

### Structure determination of GlnH.

Crystals were grown by the sitting-drop vapor diffusion method by mixing equal volumes (1 µl plus 1 µl) of protein and reservoir solution. Crystals of GlnH were grown in a mixture of 20% polyethylene glycol 6000 (PEG 6000), 0.1 M MES (morpholineethanesulfonic acid [pH 6]), and 0.2 M MgCl_2_ at 25°C with 10 mM sodium aspartate (pH 7.0), sodium glutamate (pH 7.0), or asparagine. Crystals were transferred to the reservoir solution plus 30% glycerol before being stored in liquid nitrogen.

Diffraction data were collected at Diamond Light Source and were processed with iMosflm. Phases were determined by molecular replacement with Phaser (44), using the structure of Campylobacter jejuni Asp-binding protein PEB1 as a search model (31% amino acid identity; PDB accession no. 2V25 [45]). Models were optimized using cycles of manual refinement with Coot and refinement in Refmac5 (46), part of the CCP4 software suite (47), and in Phenix (48). Data collection and refinement statistics are included in Table S1 in the supplemental material.

10.1128/mBio.00931-18.8 TABLE S1  Data collection and refinement statistics. The highest-resolution shell is shown in parentheses. Download TABLE S1, PDF file, 0.1 MB. Copyright © 2018 Bhattacharyya et al. 2018 Bhattacharyya et al. This content is distributed under the terms of the Creative Commons Attribution 4.0 International license.

Molecular graphics were generated using the PyMOL Molecular Graphics System, Version 1.7.4.0, Schrödinger, LLC (www.pymol.org).

### Trypsin digestion of GlnH.

For trypsin digestion of GlnH, 8 μg of GlnH in 10 µl phosphate-buffered saline was incubated overnight with 4 or 8 µg trypsin at 20°C with 0 or 1 mM Asp.

### Accession numbers.

Coordinates and structure factors were deposited in the Protein Data Bank with accession codes 6H1U (GlnH with Asp), 6H20 (GlnH with Asn), and 6H2T (GlnH with Glu).
