# Supplementary figures and images for: An Aspartate-Specific Solute-Binding Protein Regulates Protein Kinase G Activity To Control Glutamate Metabolism in Mycobacteria
Source: mBio. 2018 Jul 31;9(4):e00931-18. doi: 10.1128/mBio.00931-18 (PMC6069109; doi:10.1128/mBio.00931-18)

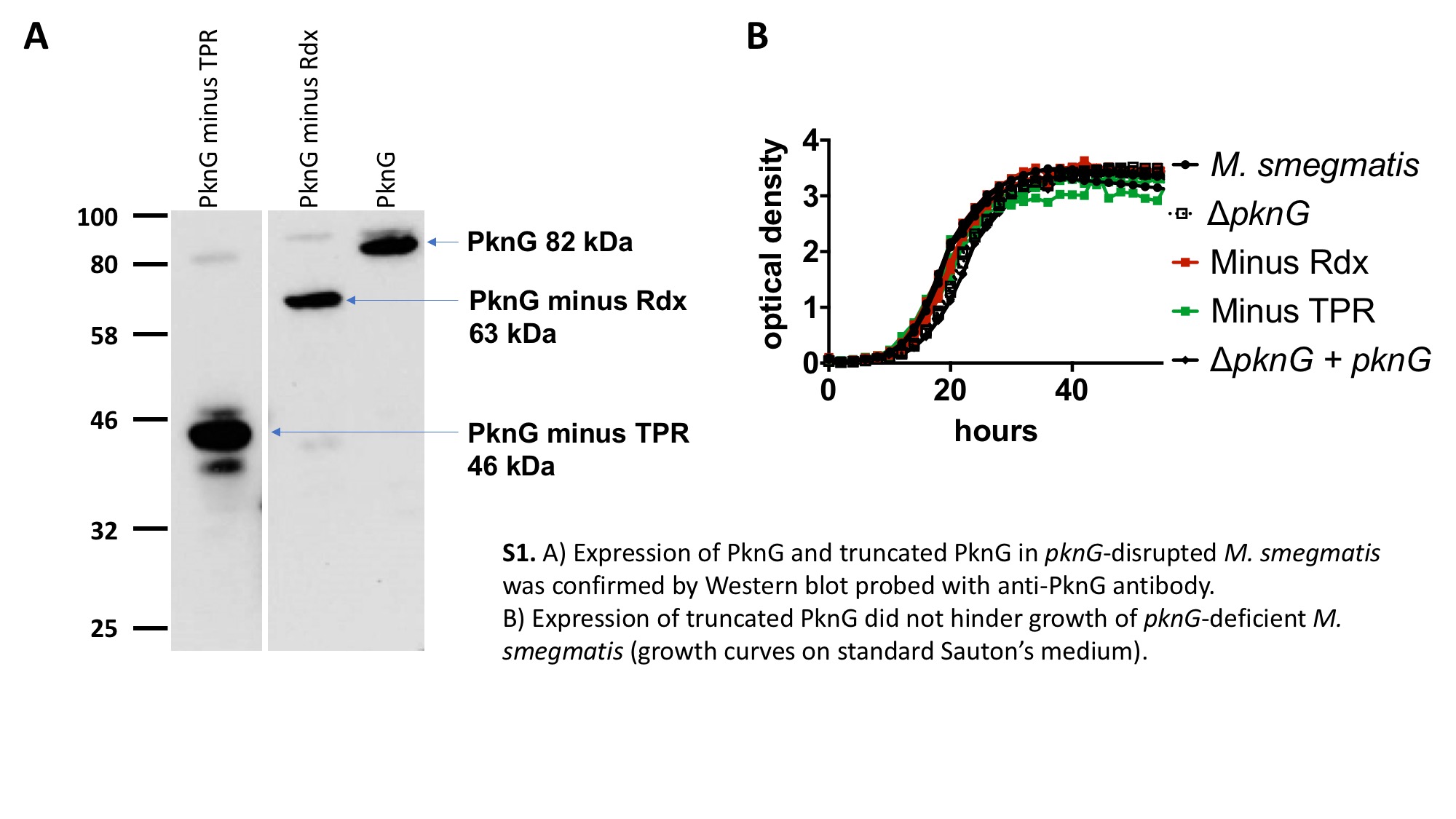

Supplement: FIG S1 [file mbo004184003sf1.jpg]

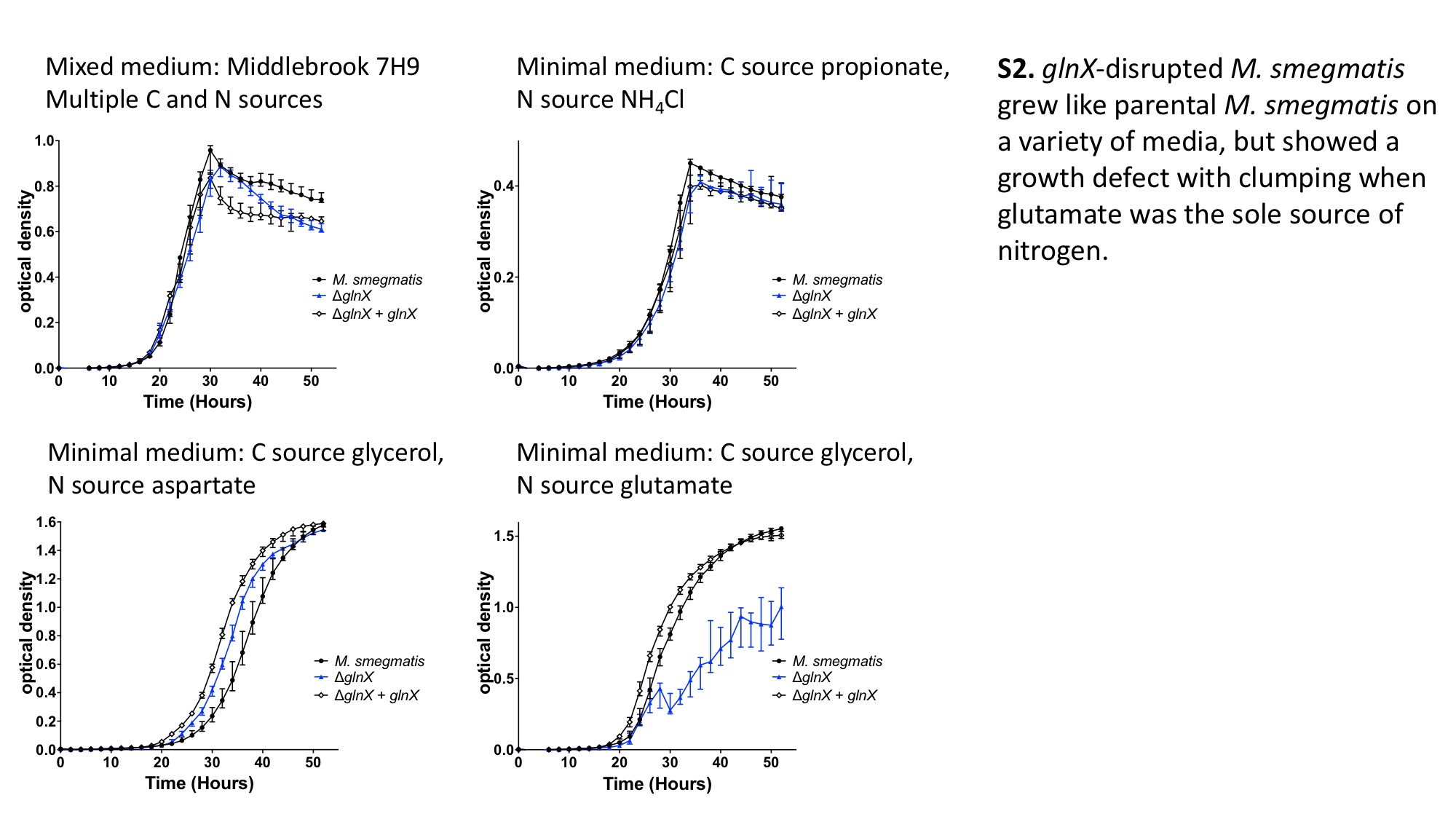

Supplement: FIG S2 [file mbo004184003sf2.jpg]

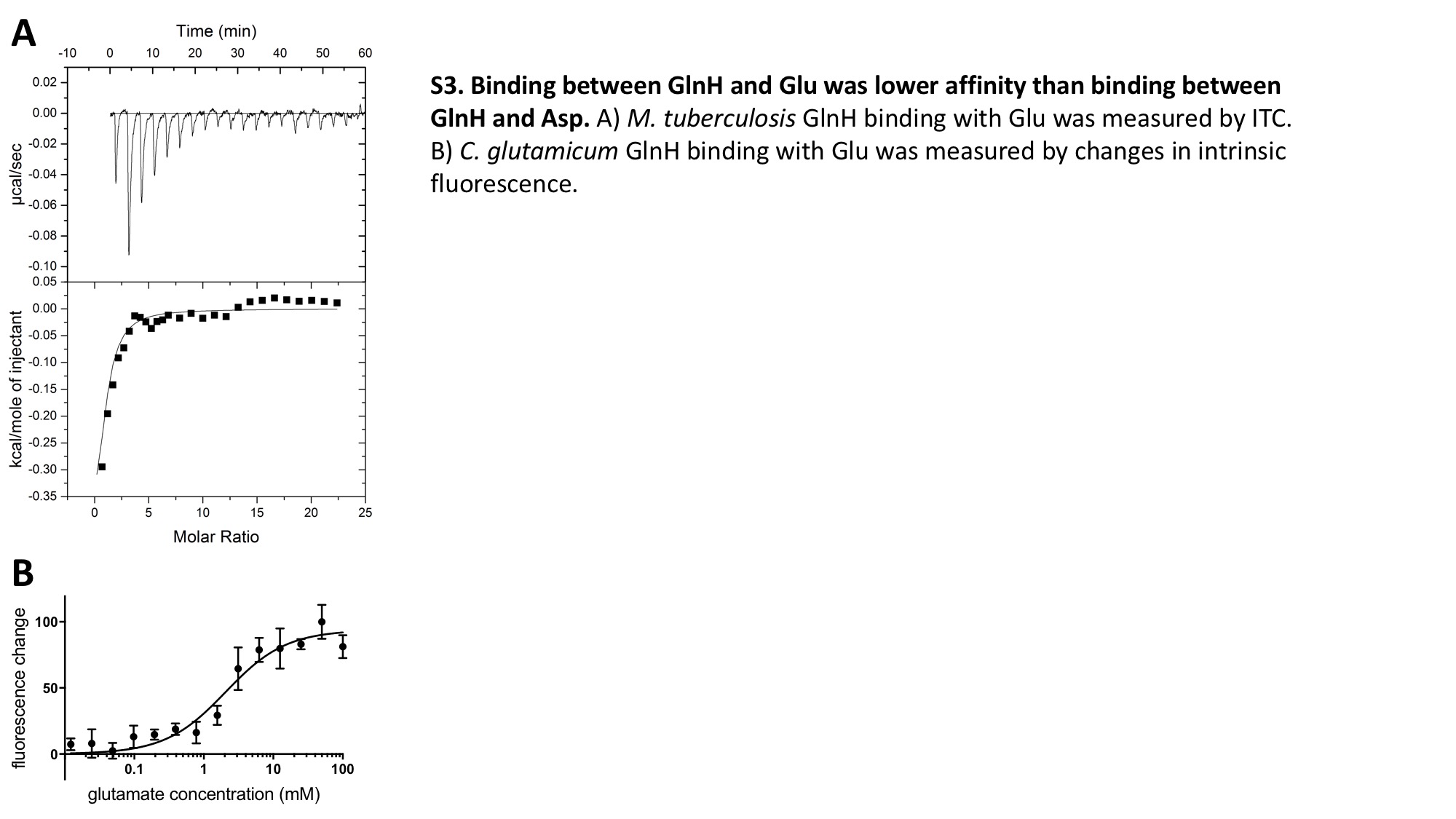

Supplement: FIG S3 [file mbo004184003sf3.jpg]

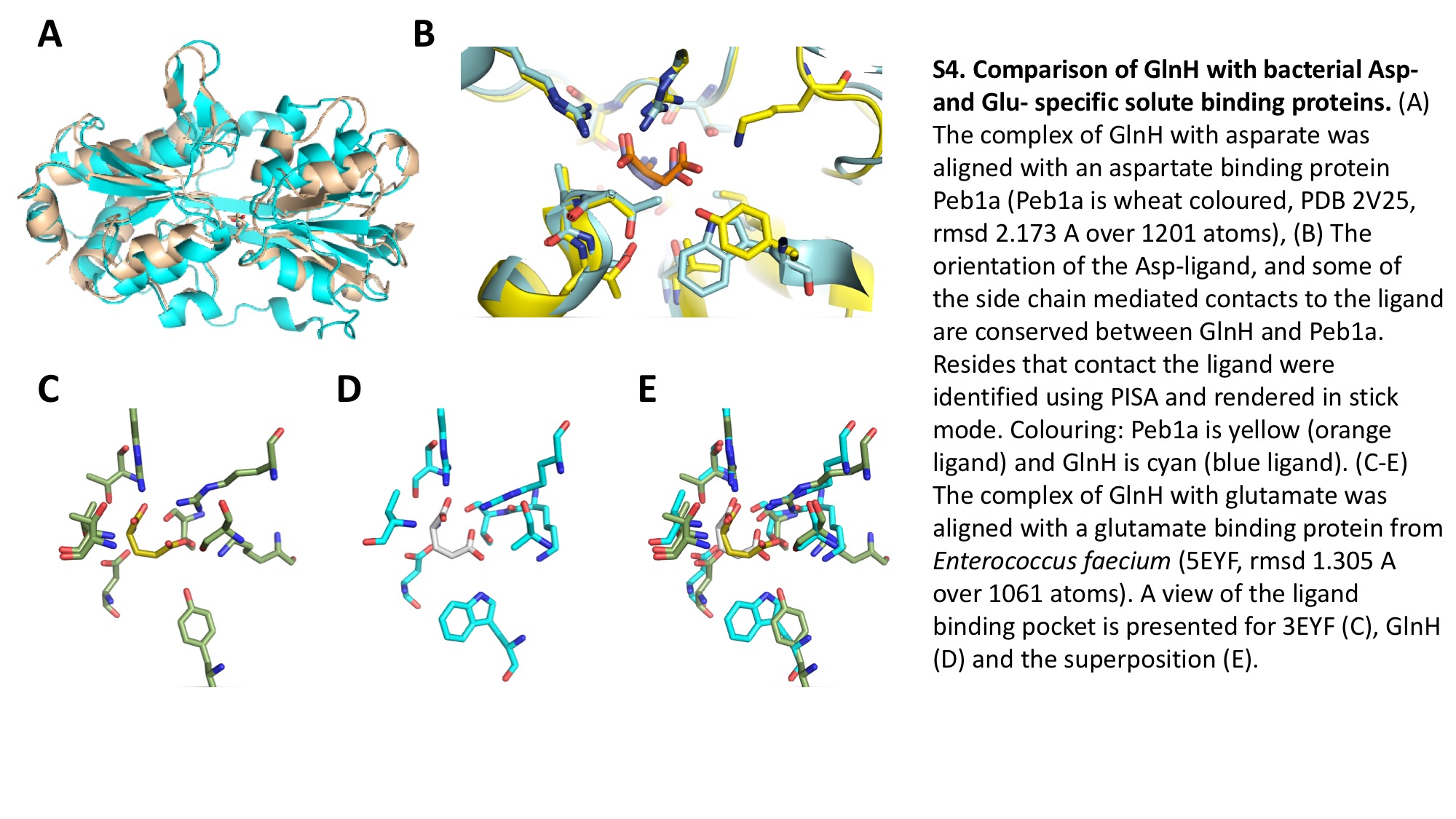

Supplement: FIG S4 [file mbo004184003sf4.jpg]

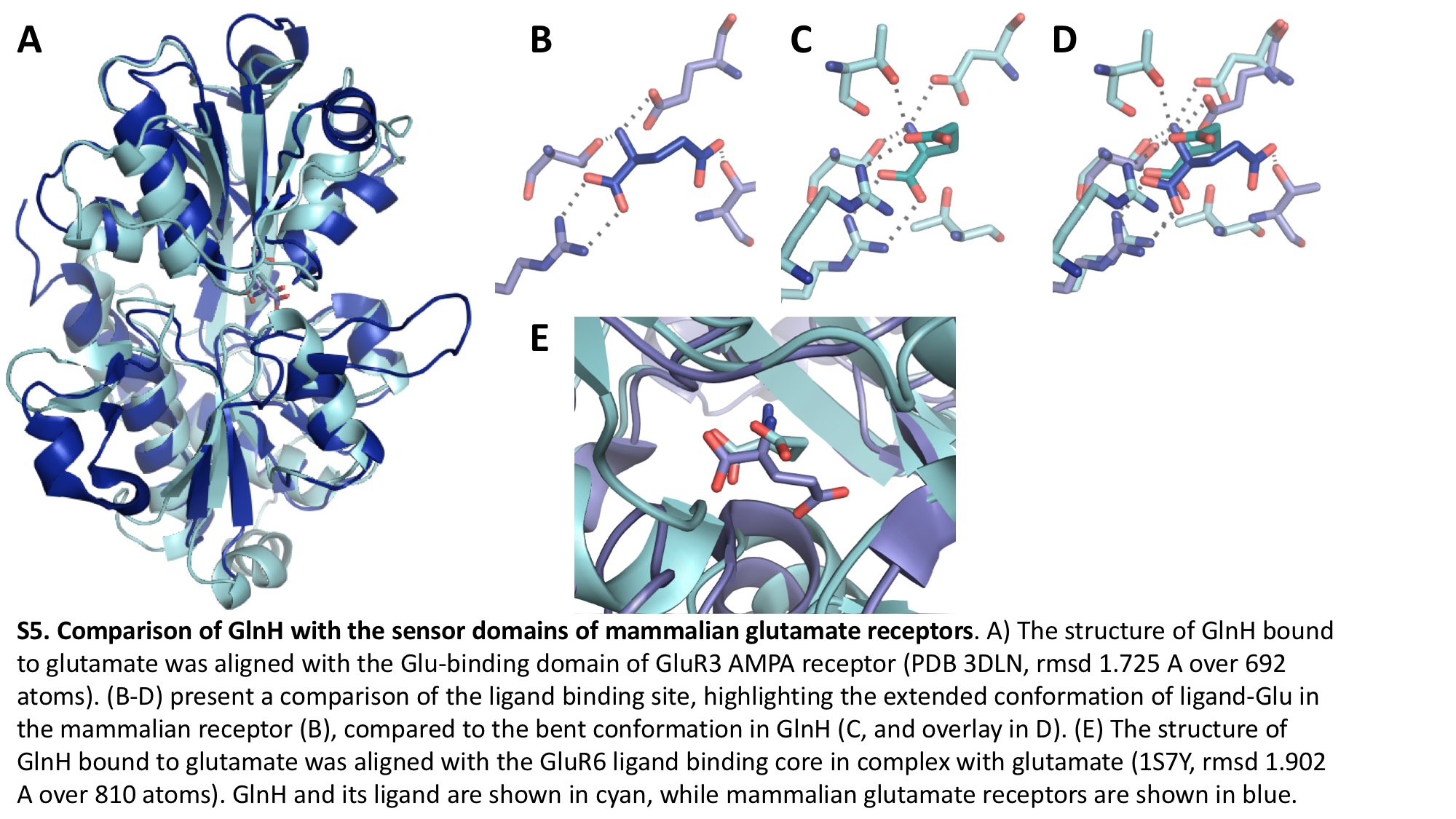

Supplement: FIG S5 [file mbo004184003sf5.jpg]

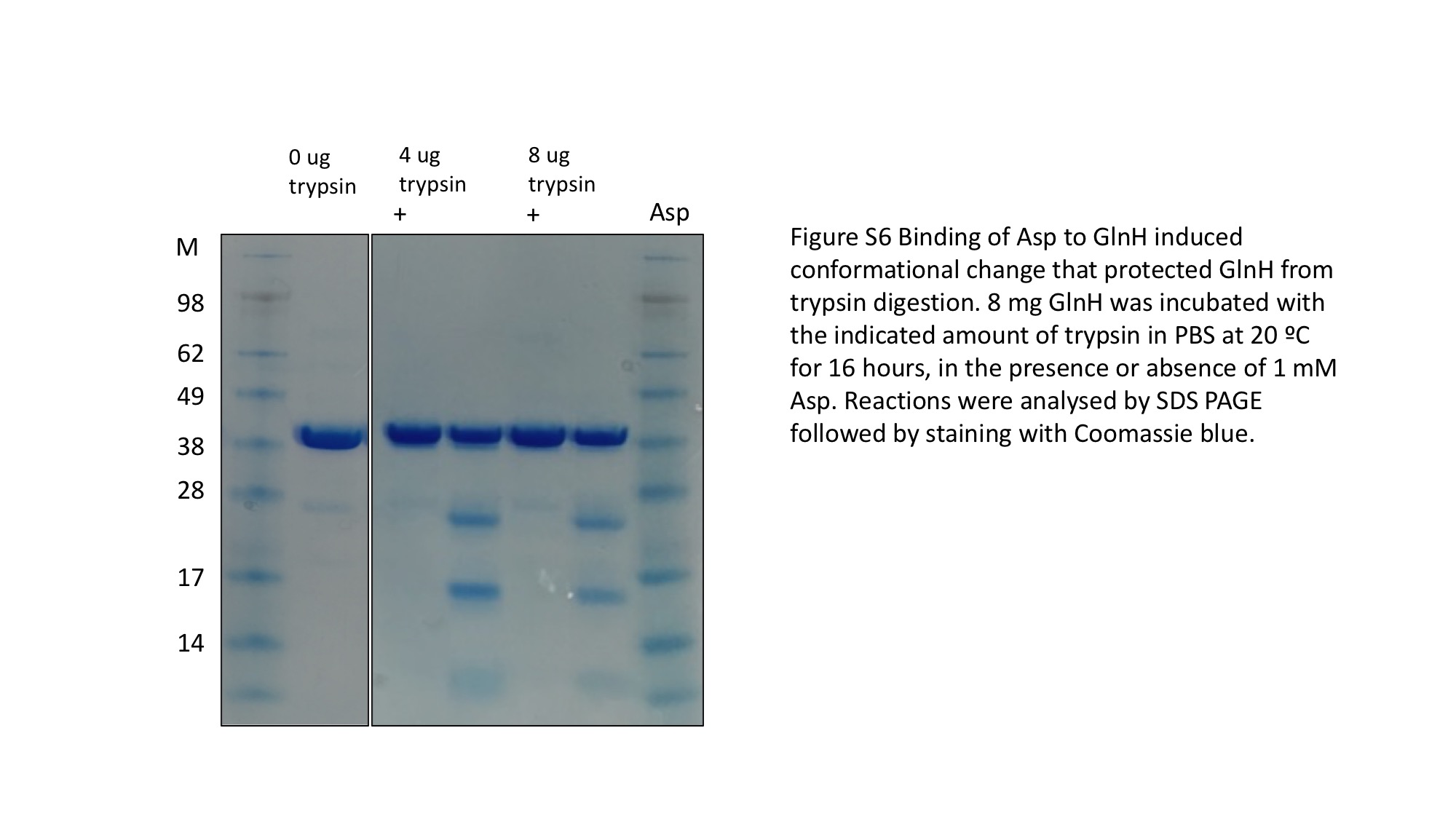

Supplement: FIG S6 [file mbo004184003sf6.jpg]

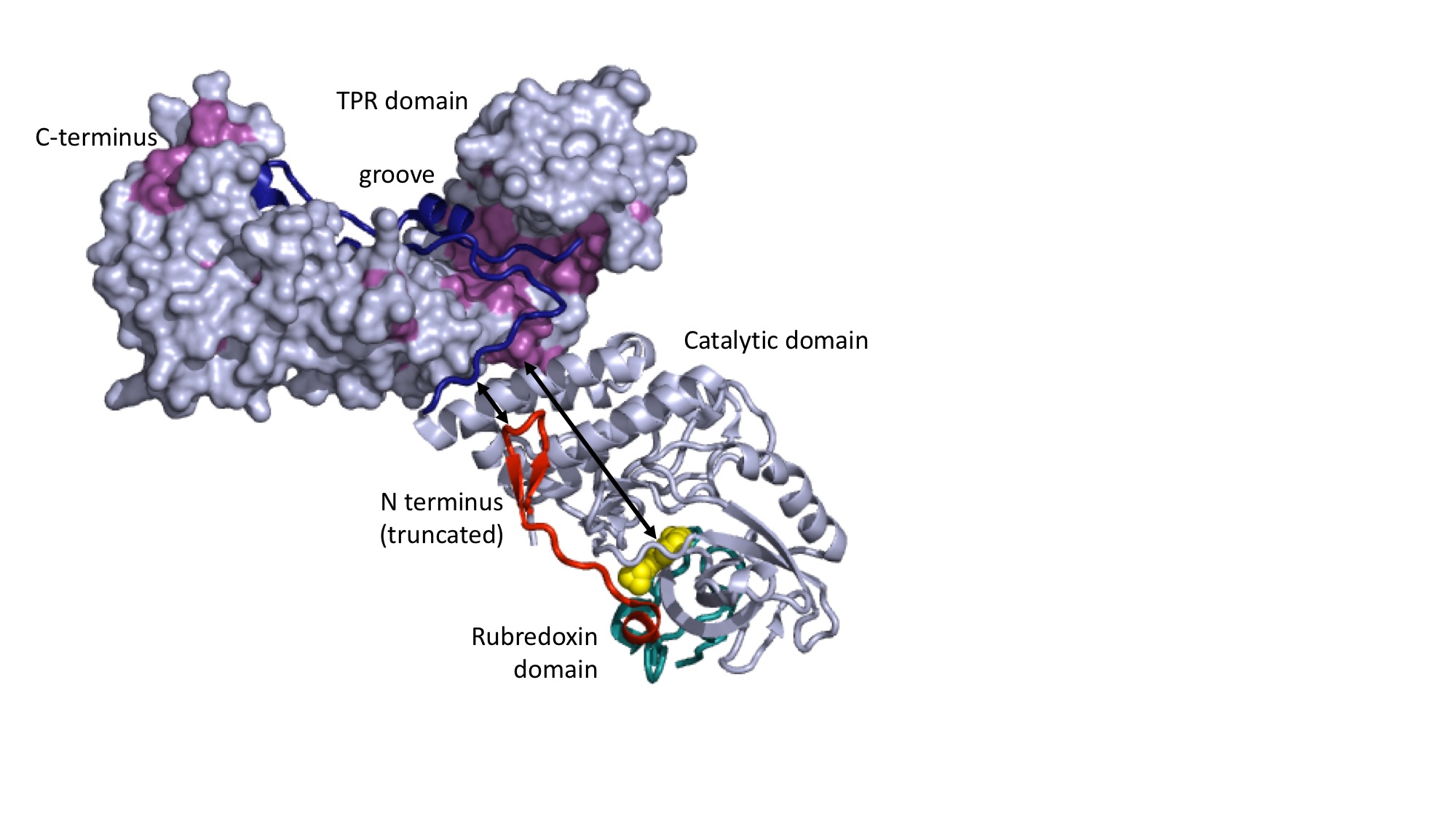

Supplement: FIG S7 [file mbo004184003sf7.jpg]
