# Supplementary material for: An Aspartate-Specific Solute-Binding Protein Regulates Protein Kinase G Activity To Control Glutamate Metabolism in Mycobacteria
Source: mBio. 2018 Jul 31;9(4):e00931-18. doi: 10.1128/mBio.00931-18 (PMC6069109; doi:10.1128/mBio.00931-18)
Supplement: TABLE S1 [file mbo004184003st1.pdf]

Table S1. Data collection and refinement statistics.

	GlnH-Asp	GlnH-Glu	GlnH-Asn
PDB code	6H1U	6H2T	6H20
Data collection			
Beam line	Diamond I04	Diamond I04	Diamond I04
Space group	$P\ 2_1\ 2_1\ 2_1$	$P\ 2_1\ 2_1\ 2_1$	$P\ 2_1\ 2_1\ 2_1$
a, b, c, Å	50.0, 81.9, 88.0	47.8, 81.1, 87.6	48.3, 81.3, 88.5
α, β, γ, º	90, 90, 90	90, 90, 90	90, 90, 90
Resolution, Å	59.93-1.68 (1.71-1.68)	59.60-1.67 (1.70-1.67)	59.87-1.4 (1.42-1.40)
R_{merge}	0.058 (0.482)	0.093 (0.607)	0.051 (0.850)
$R_{pim, \%}$	0.029 (0.236)	0.064 (0.414)	0.025 (0.410)
CC(1/2)	0.999 (0.812)	0.996 (0.543)	0.998 (0.352)
$I/\sigma I$	16.3 (3.0)	9.9 (2.6)	13.7 (1.8)
Completeness	99.95 (99.98)	99.90 (99.95)	99.84 (99.68)
Redundancy	5.9 (5.9)	5.6 (5.7)	5.8 (5.9)
Resolution, Å	59.93-1.68 (1.71-1.68)	59.60-1.67 (1.70-1.67)	59.87-1.4 (1.42-1.40)
No. reflections	241162 (12139)	226121 (11707)	400001 (20305)
R_{work}/R_{free}	0.156/0.180 (0.208/0.257)	0.158/0.178 (0.211/0.217)	0.176/0.191 (0.336/0.341)
No. atoms	2168	2573	2499
Protein	2278	2207	2209
Water	340	336	290
B-factors, Å^2	25.57	24.65	27.46
Protein	23.59	22.44	25.88
Water	38.84	38.00	39.48
Bond lengths, Å	0.009	0.019	0.008
Bond angles, º	1.13	1.45	0.95

The highest resolution shell is shown in parenthesis.
